# Compensatory Effort Parallels Midbrain Deactivation during Mental Fatigue: An fMRI Study

**DOI:** 10.1371/journal.pone.0056606

**Published:** 2013-02-14

**Authors:** Seishu Nakagawa, Motoaki Sugiura, Yuko Akitsuki, S. M. Hadi Hosseini, Yuka Kotozaki, Carlos Makoto Miyauchi, Yukihito Yomogida, Ryoichi Yokoyama, Hikaru Takeuchi, Ryuta Kawashima

**Affiliations:** 1 Department of Functional Brain Imaging, Institute of Development, Aging and Cancer, (IDAC), Tohoku University, Sendai, Japan; 2 International Research Institute of Disaster Science, Tohoku University, Sendai, Japan; 3 Smart Ageing Center, IDAC, Tohoku University, Sendai, Japan; 4 Department of Psychiatry and Behavioral Sciences, Stanford, Center for Interdisciplinary Brain Sciences Research, School of Medicine, Stanford University, Stanford, California, United States of America; 5 Japan Society for the Promotion of Science, Tokyo, Japan; Charité University Medicine Berlin, Germany

## Abstract

Fatigue reflects the functioning of our physiological negative feedback system, which prevents us from overworking. When fatigued, however, we often try to suppress this system in an effort to compensate for the resulting deterioration in performance. Previous studies have suggested that the effect of fatigue on neurovascular demand may be influenced by this compensatory effort. The primary goal of the present study was to isolate the effect of compensatory effort on neurovascular demand. Healthy male volunteers participated in a series of visual and auditory divided attention tasks that steadily increased fatigue levels for 2 hours. Functional magnetic resonance imaging scans were performed during the first and last quarter of the study (Pre and Post sessions, respectively). Tasks with low and high attentional load (Low and High conditions, respectively) were administrated in alternating blocks. We assumed that compensatory effort would be greater under the High-attentional-load condition compared with the Low-load condition. The difference was assessed during the two sessions. The effect of compensatory effort on neurovascular demand was evaluated by examining the interaction between load (High vs. Low) and time (Pre vs. Post). Significant fatigue-induced deactivation (i.e., Pre>Post) was observed in the frontal, temporal, occipital, and parietal cortices, in the cerebellum, and in the midbrain in both the High and Low conditions. The interaction was significantly greater in the High than in the Low condition in the midbrain. Neither significant fatigue-induced activation (i.e., Pre<Post), nor its interaction with factor Load, was identified. The observed midbrain deactivation ([PreH – PostH]>[PreE– PostE]) may reflect suppression of the negative feedback system that normally triggers recuperative rest to maintain homeostasis.

## Introduction

Fatigue is a physiological indication of the need for recuperative rest. It is the response of a normally functioning negative feedback system that aims to protect the body and the brain from damage due to overload. Negative feedback is a condition under which a system maintains a constant output as a result of inhibitory controls [1]. Negative feedback loops are essential for the maintenance of homeostasis. Specifically, a negative feedback system functions to protect physically fatigued muscles from excessive departures from muscle homeostasis, which could result in harm to the organism [2], [3]. Unfortunately, we often need to suppress this system to compensate for fatigue-induced performance deterioration in many everyday situations. It is often advantageous or necessary to suppress the negative feedback system at the expense of comfort, and to the cost of overworking ourselves in a competitive world, in order to rise above others in work performance or even just to achieve mediocrity. This compensatory effort may, when overexerted, lead to indefinite sick leave for organ dysfunction. In 2000, 28% of regular Japanese employees worked 50 hours or more per week. The consequence was a rise in *karoshi* (i.e., deaths due to being overworked), which reached a record high of ∼150 cases per year during 2002–2008 [4].

In this functional magnetic resonance imaging (fMRI) study, we examined the neural correlates of compensatory effort under mental fatigue. Previous functional imaging studies on fatigue have predominantly addressed the subjective feeling of mental fatigue [5]–[9]. In these studies, decreased reactivity in the lateral frontal and superior temporal cortices [7] or increased activity in the medial orbitofrontal cortex [9] during attention-demanding tasks was related to the subjective feeling of mental fatigue. These findings regarding normal functioning of the fatigue-related system are undoubtedly indispensable in understanding fatigue-related clinical states, such as chronic fatigue syndrome and *karoshi*. However, the neural correlates of compensatory effort, which are more directly related to clinical states, have not been investigated to date.

The present study focused on the neural correlates of compensatory effort because performance deterioration due to fatigue is more pronounced in tasks that demand high attention than in those that require low attention [10]. A primary challenge in identifying neural correlates of compensatory effort is to dissociate the effects on neurovascular demand from the effects of fatigue. To overcome this challenge, we assessed the effects of a fatigue intervention on the outcomes of two different task conditions that varied by degree of attentional load. After the fatigue intervention, we expected greater compensatory effort for both levels of attentional load. Therefore, we investigated whether a proportionally greater compensatory effort was required for the task requiring high attentional load than for that requiring low attentional load to maintain overall task performance.

Two distinct systems affecting different neural responses are likely to be relevant in compensatory effort. First, compensatory effort may be exerted as a function of a common top-down attention mechanism (i.e., a mechanism driven by knowledge to enhance the neuronal processing of relevant sensory input), thus facilitating discrimination between signals and distracters [11]. Among the many cortical regions involved in this mechanism, the anterior cingulate cortex (ACC) is the central controller of the mechanism [12]. Therefore, we expected the ACC and relevant sensory cortices to be activated by compensatory effort. The second mechanism is more clinically relevant. This would involve compensatory effort-induced deactivation that reflected suppression of the associated negative feedback fatigue system. There are two candidates for this system. One is the hypothalamic-pituitary-adrenal (HPA) axis, a negative feedback loop that counteracts stress. Indeed, attenuated diurnal variation in cortisol in patients with chronic fatigue syndrome [13], [14] may be a consequence of this mechanism, as it may be due to suppression of the HPA axis. The other candidate system is the striato-thalamo-cortical loop that connects the neostriatum with the prefrontal cortex [15], [16]. Attentional demands are integrated in the striato-thalamo-cortical loop, which coordinates with the dopaminergic midbrain to flexibly modulate resource allocation [17]. Chaudhuri and Behan proposed that mental fatigue is associated with striato-thalamo-cortical loop dysfunction [15].

In this study, we adopted a visual-auditory divided attention task. The task included two conditions, with low and high degrees of attentional load, in which sensory attribute differences between the target and distracters were large and small, respectively. Healthy young subjects were encouraged to maintain their level of task performance during the continuous execution of a 2-hour series of attention demanding, boring tasks. Neurovascular demand in the two levels of attentional load was measured at two time points during the study: during the first 30 minutes, following the 1-hour fatigue intervention, and during the final 30 minutes. We hypothesized that the fatigue-intervention effect on neurovascular demand would enhance the difference between attentional load conditions due to differences in the degree of compensatory-effort recruitment.

## Materials and Methods

### Ethics Statement

In accordance with the Declaration of Helsinki (1991), written informed consent was obtained from the participants prior to their participation in the present study. The Tohoku University School of Medicine Ethics Committee approved the study protocol.

### Subjects

Forty-three healthy, right-handed males (aged 20–25) from Tohoku University were recruited for the study. Individuals with perfect pitch were excluded from the study using a questionnaire because they could verbally label the pitch of the auditory stimuli used. Each participant was paid 1,000 yen per hour in compensation for their time and effort.

Before enrolment in the experiment, all participants were screened to ensure that they had no history of chronic physical or mental illness. The participants were asked to rate any subjective symptoms (scale: 1–7) that they had experienced during the previous 2 weeks, using the Japanese version of the Checklist Individual Strength Questionnaire (CIS-J) [18]. The CIS-J was used to measure chronic fatigue. The CIS is the chronic fatigue questionnaire used most frequently worldwide [19], [20]. Those with CIS-J total scores higher than 76 [18], [20] were considered probable chronic fatigue cases and were excluded. We identified four subjects with probable chronic fatigue. Thus, we enlisted 39 healthy subjects.

### Stimuli and Tasks

**Figure 1:**
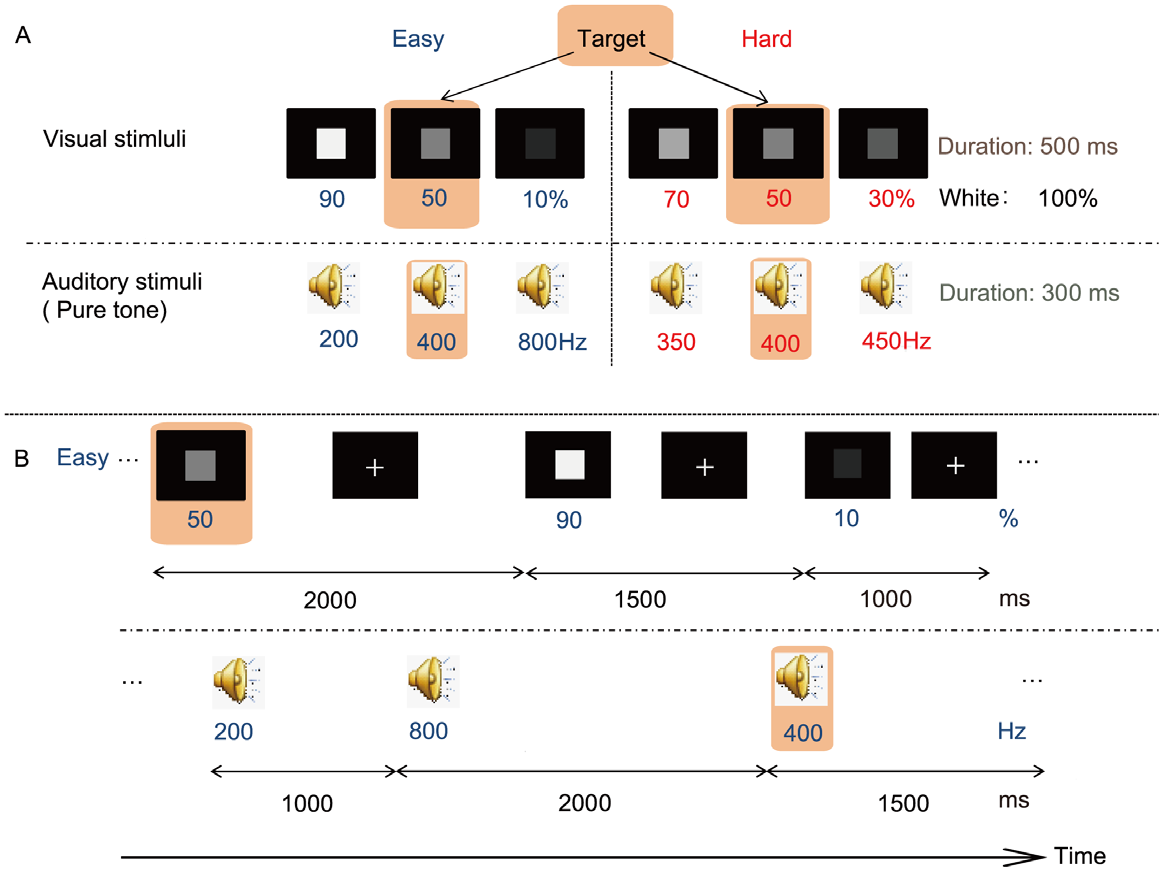
Divided attention task. A schematic of the range of visual and auditory stimuli (A) and an example showing the first three visual and auditory stimuli in an ‘easy’ sequence (B). V and A stimuli were independently presented in pseudo-random order. The V and A stimuli were presented at pseudo-random timings with stimulus onset asynchrony (SOA) of 1000, 1500, or 2000 ms. The+represents an inter-V stimulus interval, during which the screen showed a centered cross to maintain resting eye fixation. Twelve visual and 12 auditory stimuli constituted a 19-s block. A ‘hard’ block followed each ‘easy’ block. A run consisted of six easy blocks and six hard blocks, with 12-s inter-block pauses. Four sets of three runs separated by 54 s were conducted (the middle two runs were consecutive) during the 2-hour study. Subjects were instructed to press a button after each target stimulus (a different button for visual and auditory responses.).

Each subject performed a visual-auditory divided attention task comprised of alternating blocks of two different levels of attentional demand (easy [E] vs. hard [H]). The visual (V) stimuli consisted of a grey square presented for 500 ms in the center of the display and the auditory (A) stimuli consisted of a pure tone that sounded for 300 ms via a pair of MRI-compatible headphones (RTC2K MRI HEADSET, Resonance Technology, Inc., USA). All V and A stimuli were presented in pseudo-random order. Participants could clearly see the visual stimuli on a screen that was mounted on the MRI birdcage coil. The light intensity level remained the same throughout the Pre and Post sessions on the MRI scanner screen. The decibel level was optimized for each subject to ensure ease of listening and was set at the same level throughout the experiment. There were three brightness levels and three pitch levels for all stimuli. The between-level differences were smaller for the H than the E blocks. The visual stimuli brightness was 10, 50 or 90% for the E blocks and 30, 50 or 70% for the H blocks (100%: white). The pitch was 200, 400, or 800 Hz for the E and 350, 400, or 450 Hz for the H blocks. The task was to detect visual and auditory targets, which were middle-level stimuli (i.e., 50% and 400 Hz, respectively) (Figure 1). Each V or A stimulus was independently presented at a pseudo-random time with a stimulus onset asynchrony (SOA) of 1000, 1500, or 2000 ms. Participants were told in advance that the ratio of target stimuli was one-third. Each block was preceded by a 1000-ms presentation of a sample visual target at 50% brightness and a 1000-ms presentation of an “auditory target” on the screen, with a 300-ms period of a sample auditory target at 400 Hz (the total period was 4500 ms, including a 500-ms interstimulus interval between samples and a 2000-ms period of eye fixation between the end of the presentation of the sample auditory target and the start of the block). During a block, each subject was required to detect targets and respond as quickly as possible by pressing one of two buttons, using the right index finger for visual targets and the right middle finger for auditory targets. A standard block design was used. In each 19-s block, 12 visual and 12 auditory stimuli were presented. Each run consisted of six E and six H blocks. The E and H blocks were alternated and separated by a 12-s eye-fixation rest block. There was a 54-s break between the runs, which was necessary for technical reasons (i.e., response data processing and storage). Each run was preceded by two 4000-ms presentations of three sample visual stimuli (first for an E block and then an H block), plus 1000-ms inter-stimulus intervals [ISI] between the presentations. Those were followed by two 4000-ms sequences of the three sample auditory stimuli (first for an E block and then an H block). All tasks were generated using MATLAB R2008a (Mathworks, Sherborn, MA).

### Procedures

All subjects were familiarized with the task within the week prior to the experiment. Subjects were required to abstain from drinking alcohol 48 hours before and caffeinated beverages 24 hours before the experiment. Alcohol intake was associated with acute increases in cerebral blood flow, particularly in the frontal regions [21]. Furthermore, there was an effect of caffeine on the fronto–parietal network, which is involved in top-down cognitive control during encoding. Moreover, an effect was also observed on the prefrontal cortico–thalamic loop, which is involved in the interaction between arousal and top-down control of attention during maintenance [22]. They were required to have a meal before the experiment. We conducted all experiments during normal studying hours so that circadian rhythms were not disturbed. Subjects were not informed of the number of runs that they would perform nor allowed to see a watch or a clock during the experiment to prevent any motivational changes that might occur from expecting the task to end soon.

Each subject performed twelve runs during the experiment. The first three runs in the MRI scanner comprised the “Pre” session. Six subsequent runs outside the scanner in a soundproof room comprised the “fatigue intervention” session. The last three runs, again in the MRI scanner, comprised the “Post” session.

Before the experiment began on the experimental day, the subjects practiced two runs to familiarize themselves with the task. After taking a 15- to 30-minute break, each subject was laid in the fMRI scanner. Head motion was minimized using foam pads and a headband. Participants viewed the visual stimuli with a projector (DLA-HD10KS, Victor, Inc., Japan), which back-projected images onto a screen (MR-VF01, Kiyohara Optics, Inc., Japan) attached to the head coil of the MRI. Pure tones were heard via a pair of MRI-compatible headphones that were set at a comfortable volume for the subjects. Behavioral responses were recorded using a two-button fiber-optic response box (Current Designs, Inc., Philadelphia, PA).

At the beginning and end of each run, the subjects self-evaluated their level of fatigue, aversion to continuing the divided attention task, and sleepiness using an 11-grade scale (0: “not at all” to 10: “maximum”). Additionally, to evaluate the degree of compensatory effort exerted, subjects self-evaluated the degree of task difficulty separately for the E and H blocks at the end of each run. After this self-evaluation, subjects had a 54-s break between the runs.

The length of the Pre, fatigue-intervention, and Post sessions were 27 min 58 s, 56 min 12 s, and 27 min 58 s, respectively. Subjects spent no more than 10 min moving between the fMRI scanner and a soundproof room. The length and number of the runs were optimized by preliminary behavioral studies so that most of the subjects felt fatigued but did not retire due to the task execution.

### Image acquisition

All MRI data was acquired with a 3-T MRI scanner (Philips Achieva Quasar Dual, Philips Medical Systems, Best, The Netherlands). Functional images were obtained using an echo-planer image (EPI) pulse sequence. Forty transaxial gradient-echo images (echo time = 30 ms, flip angle = 80°, slice thickness = 2.5 mm, FOV = 192 mm, matrix = 64×64) covering the entire brain were acquired at a repetition time of 2.5 s. For each run, 176 EPI scans were acquired excluding three dummy scans for stabilization of the T1-saturation effect.

### Functional imaging data analysis

Pre-processing and analyses of fMRI data were performed using Statistical Parametric Mapping software (SPM5; Wellcome Department of Cognitive Neurology, London, UK) implemented on MATLAB R2008a (Mathworks Inc., Natick, MA, USA). For the pre-processing analyses, images were corrected for slice timing and head motion in each subject (i.e., the first image was used as a reference to which all subsequent scans in all runs was realigned). Hence, none of the registrations of a subject's images differed between the Pre and Post sessions. Images were then spatially normalized to the EPI-MNI template and spatially smoothed using an 8-mm full-width at half-maximum (FWHM) Gaussian kernel.

A standard two-stage approach was employed for statistical analysis. As a first-level analysis, the degree of activation or deactivation from resting condition was estimated for each subject using a general linear model (GLM) framework. Pre-processed images of the Pre and Post sessions (i.e., six runs) and four hemodynamic models (i.e., for PreE, PreH, PostE, and PostH conditions; reference vector: PreE, PreH, PostE, PostH) were constructed using the standard hemodynamic function supplied by SPM5, which represents the standard BOLD response caused by brief neurovascular demand [23]. For the second-level analysis, between-condition statistical inference was executed on a two-by-two factorial model that included the parameter-estimate images of the four conditions from all the subjects. That is, the model was comprised of the factor load (i.e., E vs. H) and time (i.e., Pre vs. Post). Although the model conformed to the two-way repeated measure analysis of variance (ANOVA), *t*-tests were used instead of *F*-tests because we wanted to identify neurovascular demand based on *a priori* hypotheses.

First, the effect of fatigue was examined separately in two conditions. Fatigue-induced activation in the H (PostH>PreH) and E (PostE>PreE) conditions was identified using the contrasts PostH – PreH (reference vector: 0 −1 0 1), and PostE – PreE (reference vector: −1 0 1 0), respectively. Fatigue-induced deactivation under the H (PreH>PostH) and E (PreE>PostE) conditions was identified using the contrasts PreH – PostH (reference vector: 0 1 0 −1) and PreE – PostE (reference vector: 1 0 −1 0), respectively. Then, we searched for a possible effect of compensatory effort. Activation due to compensatory effort was identified by contrasting fatigue-induced activation for the E against the H condition, (i.e., [Post H – PreH] – [PostE – PreE]; reference vector: 1 −1 −1 1). This analysis was confined to the areas where fatigue-induced activation was significant (i.e., inclusively masked by the contrast PostH – PreH at a liberal threshold of *p*<0.05, uncorrected). Similarly, deactivation due to compensatory effort was identified by ([Pre H – PostH] – [PreE – PostE]; reference vector: −1 1 1 −1), inclusively masked by the (PreH – PostH) contrast. All the main contrasts were thresholded at the family-wise error (FWE) rate of *p*<0.05 and corrected for multiple comparisons, assuming the whole brain as the search volume using random field theory (RFT), and defining theoretical results for smooth statistical maps at a false-positive rate of <5% in the searching area.

## Results

### Behavioral data

Performance accuracy was evaluated as the mean of hit and correct-rejection rates; visual and auditory stimuli responses were pooled. Seven participants whose mean accuracy across the Pre session was less than 75% (chance level: 50%) were excluded based on the assumption that they were already fatigued or were unaccustomed to the fMRI environment. Three participants were also excluded due to trouble with their technique. Therefore, we analyzed data from 29 subjects (mean ± SD age: 21.4±1.2 years).

**Figure 2:**
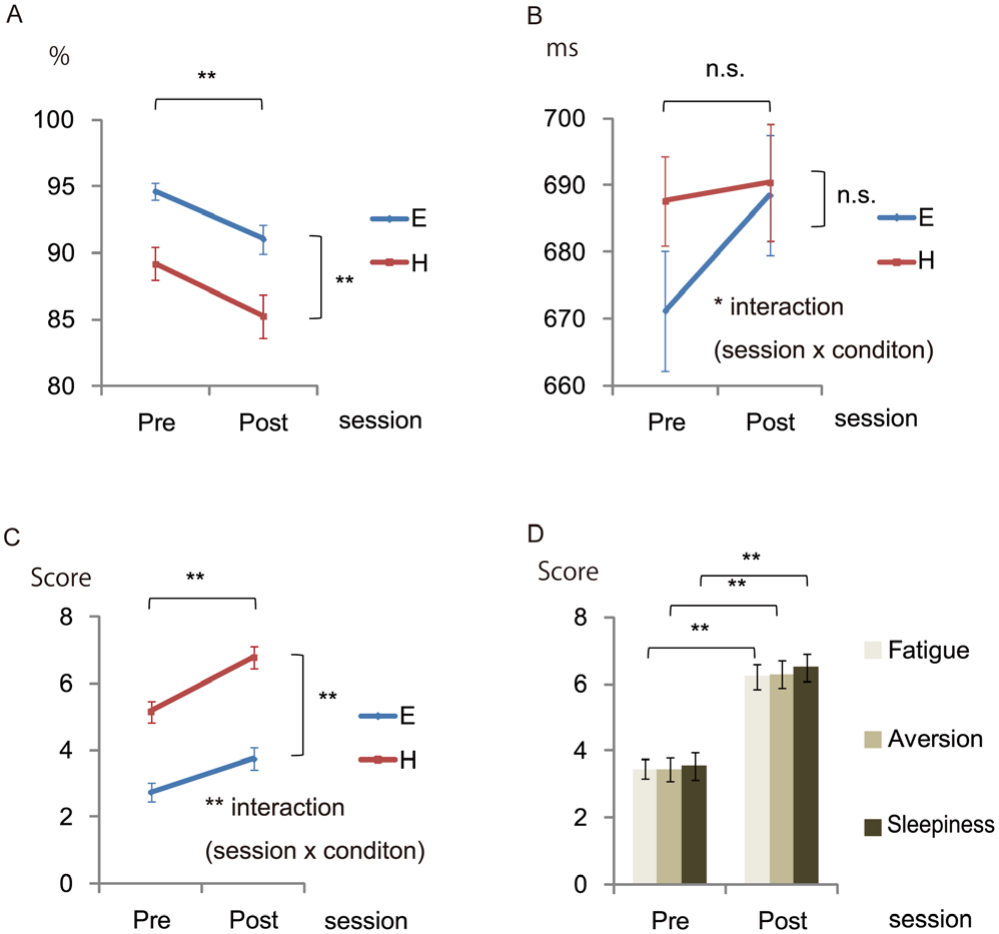
Behavioral data (n = 29). The mean percent accuracy (A) and mean reaction times (B) in both Pre and Post sessions during functional MRI scanning are shown for the easy and hard conditions. Each line indicates the subjective task difficulty of the easy and hard tasks, which were analyzed using a two-way ANOVA for factors Load (Easy vs. Hard) and Time (Pre vs. Post) (C). Each bar graph indicates the means of the subjective feelings of fatigue, aversion, and sleepiness in both Pre and Post sessions, which were analyzed using paired *t*-tests (D). Error bars indicate standard errors. * *p*<0.05; ** *p*<0.001. E, Easy; H, Hard; n.s., no significance; Post, the last three runs again in the magnetic resonance imaging (MRI) scanner; Pre, the first three runs in the MRI scanner.

The behavioral data are summarized in Figure 2. The mean accuracy and reaction times for correct responses, as well as subjective task-difficulty, were analyzed using a two-way repeated measure ANOVA. For the mean accuracy, the main effect of Load (*F* [1, 28] = 41.8, *p*<0.001) and Time (*F* [1, 28] = 20.1, *p*<0.001) was significant, but the interaction was not (Figure 2A). For mean reaction time, only the Load×Time interaction (*F* [1, 28] = 6.0, *p* = 0.021) was significant (Figure 2B). For subjective task difficulty, the main effect of Load (*F* [1, 28] = 128.4, *p*<0.001), Time (*F* [1, 28] = 47.6, *p*<0.001), and the Load×Time interaction (*F* [1, 28] = 18.0, *p*<0.001) were significant (Figure 2C).

Other psychological measures, namely fatigue, aversion, and sleepiness, were analyzed using paired t-tests. Significant increases in fatigue (*t* [28] = 8.5, *p*<0.001), aversion (*t* [28] = 8.8, *p*<0.001), and sleepiness (*t* [28] = 6.6, *p*<0.001) was observed (Figure 2D).

### fMRI data

**Figure 3:**
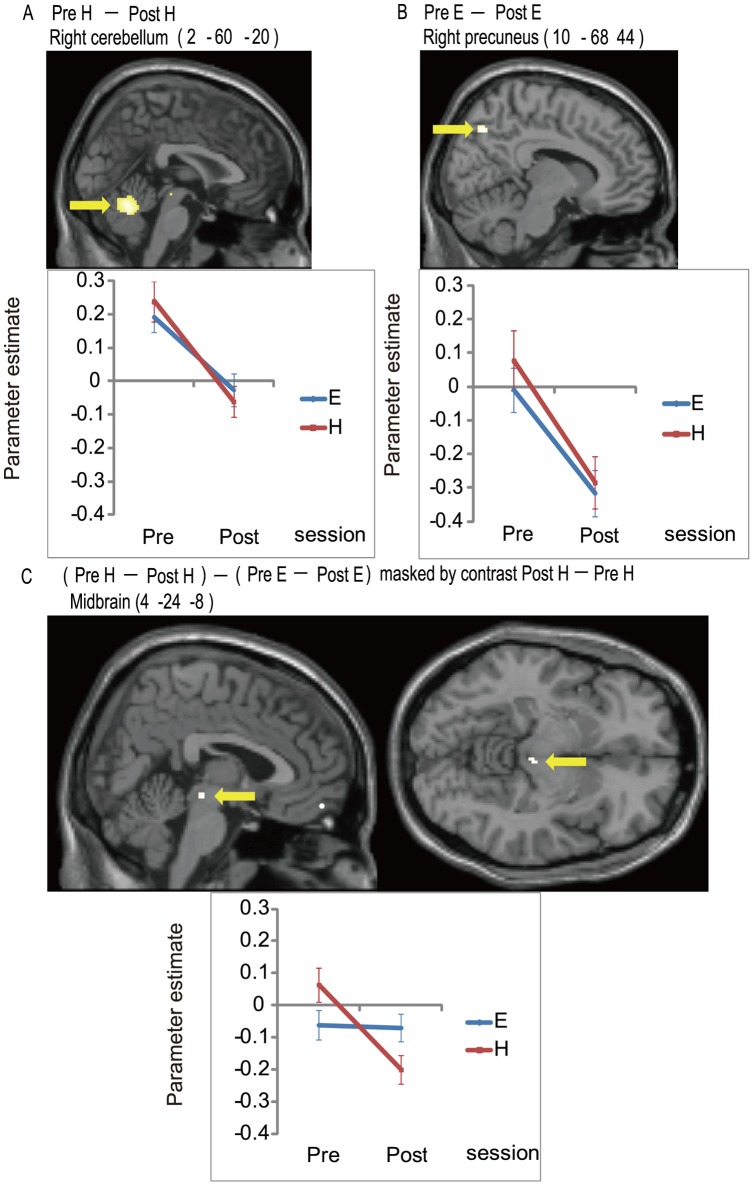
Deactivation related to fatigue and compensatory effort (n = 29). All voxels were significant at a statistical threshold of *p*<0.05 for family wise error (FWE) corrected for multiple comparisons. Fatigue-induced deactivation in the H condition (i.e., PreH – PostH) (A), fatigue-induced deactivation in the E condition (i.e., PreE – PostE) (B), and deactivation reflecting the compensatory effort (i.e., [PreH – PostH] – [PreE – PostE]) (C). The activation profile of each area represents the parameter estimates in each condition. Errors bar represent the standard errors. The coordinates in the MNI standard space are indicated. E, Easy; H, Hard; Post, the last three runs again in the magnetic resonance imaging (MRI) scanner; Pre, the first three runs in the MRI scanner.

**Figure 4:**
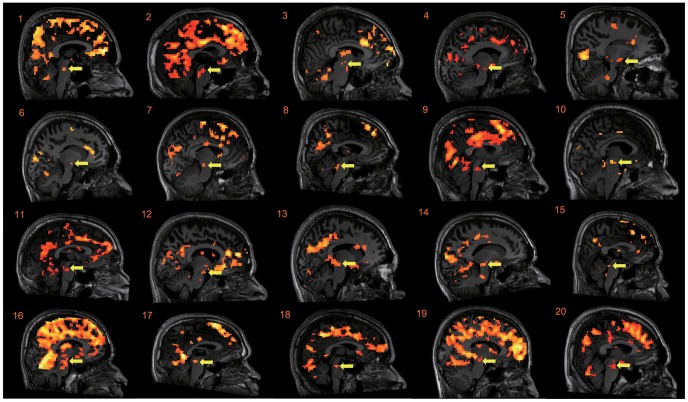
Individual analyses. Deactivation in the midbrain reflecting compensatory effort (i.e., [Pre H – PostH] – [PreE – PostE]) was re-analyzed on an individual basis within the subject's native space, and each anatomical image was overlain on the mid-sagittal section. All voxels were significant at a statistical threshold of *p*<0.05, uncorrected.

The fMRI data are summarized in Table 1 and Figure 3. Neither significant fatigue-induced activation ([PostH>PreH] and [PostE>PreE]) nor activation related to compensatory effort ([Post H – PreH]>[PostE – PreE]) was identified. In the H condition (i.e., PreH – PostH), significant fatigue-induced deactivation (PreH>PostH) was identified in the right superior frontal gyrus, bilateral middle frontal gyrus, left superior temporal gyrus, left middle occipital gyrus, right fusiform gyrus, right precuneus, right posterior lobe of the cerebellum, and midbrain. In the E condition (i.e., PreE – PostE), significant fatigue-induced deactivation (PreE>PostE) was identified in the left middle frontal gyrus, right inferior temporal gyrus, right postcentral gyrus, left middle occipital gyrus, right angular gyrus, right precuneus, and right posterior lobe of the cerebellum. Significant deactivation relevant to compensatory effort (i.e., [PreH– PostH]>[PreE – PostE]) was identified in the midbrain. The deactivation appeared to be located near the periaqueductal gray matter (PAG) and cuneiform (CnF) of the reticular activation formation [24]. Furthermore, because the midbrain contains small anatomical structures, a concern about whether the observed differences were due to an inappropriate normalization of standardized space was raised [25]. Hence, the analysis was also conducted on an individual basis within the subject's native space, and each anatomical image was overlain on the mid-sagittal section. Analysis of the fMRI images showed a significant (*p*<0.05, uncorrected) difference in the midbrain regions in 20 of 29 subjects (Figure 4).

**Table 1:** Deactivation related to fatigue and compensatory effort (n = 29).

		PreH – PostH (Fatigue)		PreE – PostE (Fatigue)		(PreH – PostH) – (PreE – PostE) (Compensatory effort)	
				
Brain region	side	MNI Coordinate	z Score	MNI Coordinate	z Score	MNI Coordinate	z Score
Superior frontal gyrus	R	(20, 4, 72)	4.80				
Middle frontal gyrus	R	(38, 2, 62)	5.38				
		(24, 4, 50)	5.01				
		(54, 16, 42)	4.97				
		(58, 22, 28)	4.95				
	L	(−60, −64, 0)	4.88	(−58, −64, 2)	4.77		
Superior temporal gyrus	L	(−58, −34, 6)	5.03				
Inferior temporal gyrus	R			(52, −68, −8)	4.93		
Postcentral gyrus	R			(46, −36, 56)	4.83		
Middle occipital gyrus	L	(−54, −68, −16)	5.78	(−52, −64, −18)	4.86		
Fusiform gyrus	R	(46, −60, −26)	5.50				
		(48, −24, −20)	4.97				
Angular gyrus	R			(34, −62, 40)	4.85		
Precuneus	R	(10, −70, 42)	6.05	(10, −68, 44)	5.12		
		(32, −70, 38)	5.35				
Posterior lobe of cerebellum	R	(2, −60, −20)	5.88	(4, −62, −20)	5.17		
Midbrain		(8, −26, −10)	5.61			(4, −24, −8)	4.85

E, Easy; H, Hard; L, left; MNI, Montreal Neurological Institute; Post, the last three runs again in the magnetic resonance imaging (MRI) scanner; Pre, the first three runs in the MRI scanner; R, right.

## Discussion

To the best of our knowledge, this is the first study to demonstrate the neural correlates of compensatory effort under acute mental fatigue. Midbrain deactivation after fatigue intervention (Pre>Post) was more prominent in the high (PreH≫PostH), as compared to the low (PreE>PostE) attention-demanding condition. This deactivation pattern was expected for the negative feedback homeostasis system related to fatigue, the function of which was suppressed during compensatory effort.

Behavioral data showed that compensatory effort was indeed recruited more in the H than in the E condition after the fatigue intervention. Although most of the behavioral data consistently showed an effect of the fatigue intervention, the mean accuracy did not show a larger deficit under the H condition (Figure 2A), which was expected given the absence of compensatory effort [16], [26]. Furthermore, the mean reaction time in the H condition was maintained after the intervention, in contrast to the prolonged reaction time in the E condition, as is reflected in the significant Load×Time interaction (Figure 2B). This observation seems to be evidence of performance over-compensation in the H condition. The increase in subjective task-difficulty was larger in the H than in the E condition, probably reflecting compensatory effort.

Midbrain involvement in fatigue-related processes has commonly been postulated. Attentional demands are integrated in the striato–thalamo–cortical loop, which coordinates with the dopaminergic midbrain to flexibly modulate resource allocation [17]. Interruption of this loop to suppresses cortical activation predisposes one to fatigue symptoms [15]. The striato–thalamo–cortical loop architecture has been proposed to also extend to closed-loop subcortical connections and brainstem sensorimotor structures, including the PAG and CnF [27]. A brainstem fatigue-generator model has also been postulated in post-poliomyelitis fatigue and post-viral fatigue syndromes [28]. Post-mortem histopathology in acute poliomyelitis with severe fatigue revealed poliovirus lesions in the midbrain [29].

Several lines of evidence support the notion that the observed midbrain deactivation ([PreH – PostH]>[PreE – PostE]) reflects the suppression of the negative feedback homeostasis system. First, midbrain involvement is highly probable in the fatigue-related negative feedback system, considering its critical contribution in controlling energy homeostasis [30]. Midbrain dopaminergic neurons sustain important physiological functions by motivating behavior thorough coordination of neuroendocrine, behavioral, and metabolic effectors of energy balance [31]. Indeed, a previous study demonstrated that a long-loop negative feedback control of dopamine neurons from the nucleus accumbens (ACB) to the ventral tegmental area in the midbrain required cooperative interaction of dopamine 1 and dopamine 2 receptors in the ACB of rats [32]. Second, there is evidence that suppression of the negative feedback homeostasis system is a result of midbrain dysfunction. Conditional gene knockdown of the leptin receptor by a virus injection into the midbrain ventral tegmental area resulted in overeating in rats [33]. Furthermore, there is clinical evidence that prolonged compensatory effort may affect the midbrain, both anatomically and functionally. A decrease in midbrain white matter volume was observed with increasing fatigue duration in patients with chronic fatigue syndrome [34] and was suggested to influence both peripheral and central homeostasis. Disrupted peripheral homeostasis was demonstrated as an abnormal relationship between MRI levels in the CnF and peripheral pulse pressure [34]. Taken together, it appears reasonable to hypothesize that compensatory effort under mental fatigue is, at least in part, exerted by suppression of the negative feedback homeostasis system in the midbrain.

Conversely, some may suspect reduced motivation as a possible explanation for the observed midbrain deactivation in the H condition (PreH>PostH). However, this interpretation is not supported by our behavioral data; the increase in reaction time was less under the H condition than under the E condition (Figure 2B). One can also question whether mental load induces higher levels of not only compensatory effort but also fatigue itself with its negative feedback effects. However, fatigue is a condition that develops (from the cumulative effect [18], [20], [35] of the E and H tasks) more slowly than the delay between the E and H tasks. That is, the fatigue intervention lasted 1 hour, compared with 35.5 seconds between the start of alternating E and H blocks. Therefore, the cumulative fatigue effect was the same under consecutive E and H conditions.

One may note that the midbrain showed greater activation in the H than in the E condition in our Pre session, which cannot be explained as an effect of compensatory effort. The effect of attentional demand *per se* may account for this observation. It has been established that midbrain activation is enhanced during attention-demanding tasks [17], [36], [37]. Therefore, the observed activation pattern (PreH from rest condition) in the midbrain may be explained as follows. The facilitative effect of attentional demand predominated in the Pre session, whereas this effect was overtaken by the suppressive effect of compensatory effort in the Post session.

Here, we briefly discuss the fatigue-induced deactivation (for the H [PreH>PostH] and E [PreE>PostE]) observed in several cortical areas, including the middle frontal gyrus, medial precuneus, and right posterior lobe of the cerebellum. The observed deactivation can be explained by time or order effects, including fatigue. Deactivation of these regions has been reported during tasks intended for motor-sequence learning [38], [39]. Another candidate concept for explaining the observation is task-induced deactivation (TID), which refers to deactivation during any attention-demanding task [40]. TID increased during a difficult task [40], which may explain the enhanced deactivation in our Post session due to increased subjective task difficulty.

We found no significant fatigue-induced activation ([PostH>PreH] and [PostE>PreE]). This observation may be surprising in light of the established involvement of many cortical regions during top-down attention [12], [41]. This finding might, however, stem from an implicit assumption that the suppressive effect should be exerted by a mechanism related to top-down attention. Our findings suggest that this assumption may not be true. The degree of top-down attention for task execution may be constant across the Pre and Post sessions, while the suppressive compensatory effect is exerted independently from top-down attention.

The current finding has significant clinical implications. Understanding the mechanism by which compensatory effort under mental fatigue damages homeostasis impacts public health policy. That is, the mechanism would clearly demonstrate that the consequence of excessive compensatory effort or overwork could become somatic. However, it is important to note that the current finding is only indirect evidence of this notion, and the findings were derived from subjects of a limited sex/age group using a specific cognitive task. Several lines of confirmatory research must follow, such as a similar experiment using different sex or age groups, different tasks, or experimental suppression of the midbrain in animals under fatigue.

## Conclusions

We demonstrated that fatigue-induced deactivation (Pre>Post) in the midbrain was greater in the high (PreH≫PostH) than in the low (PreE>PostE) attention-demanding condition. Recruitment of compensatory effort during fatigue was also confirmed by behavioral data. These observations may be explained by the hypothesis that compensatory effort is affected by a mechanism that suppresses the negative feedback system that normally triggers recuperative rest to maintain homeostasis.
